# Impact of Sociodemographic Factors on Use of Formal Social Services in an Older Swedish Population

**DOI:** 10.3390/ijerph191912526

**Published:** 2022-09-30

**Authors:** Emilie Stroh, Anna Axmon, Connie Lethin, Gunilla Carlsson, Agneta Malmgren Fänge, Kristoffer Mattisson

**Affiliations:** 1 Division of Occupational and Environmental Medicine, Department of Laboratory Medicine, Environment, Society & Health, Lund University, SE-221 00 Lund, Sweden; 2 EPI@LUND (Epidemiology, Population Studies, and Infrastructures), Lund University, SE-221 00 Lund, Sweden; 3 Department of Health Sciences, Lund University, SE-221 00 Lund, Sweden

**Keywords:** community and health care, healthy aging, home care services, informal care, institutional long-term care, nursing home, social services, sociodemographic factors

## Abstract

Background: In Sweden, societal support for older people is the responsibility of the municipalities. However, due to Sweden’s current aging-in-place policy for older people, there is a need to assess how the use of such services varies based on sociodemographic factors. The aim of this study was to describe the use of different forms of social services and institutional long-term care (ILTC) in an older population and to evaluate the impact of sociodemographic factors. Methods: This was a cross-sectional register-based study, including all individuals aged 65 years and older in two Swedish municipalities in 2010, 2015, and 2019. The study analyzed the use of social services and ILTC in relation to sex, place of birth, cohabitation status, and type of housing. Results: Women, those born in Sweden, and those living in an apartment were more likely to receive assistance than men, those born abroad, or living in single family houses, respectively. People living alone were consistently more likely to have assistance, as well as ILTC. Conclusions: There may be a discrepancy between the individual’s need and the assistance provided from the municipality in certain sociodemographic groups in the older population in Sweden.

## 1. Introduction

In Sweden, as well as worldwide, the population increasingly tends to live longer. Between 2002 and 2019, the life expectancy from birth increased in Europe (from 80.9 to 84.0 years for women, and from 74.3 to 78.5 years for men) [1]. In Sweden, people aged 65 years and older had similar increase in life expectancy, i.e., about four years, during the past decades. 

In Sweden, care for the old is a part of the welfare system. The Swedish aging-in-place policy implies that older people should live in their own housing for as long as possible and be provided with the necessary social services to do so. This policy relies on the Social Services Act [2], which regulates the services and support aimed at those needing it to be able to live an independent life in their own home. The aim of social services for older people is to enable a dignified and good life. It is the responsibility of the National Board of Health and Welfare, a government agency under the Ministry of Health and Social Affairs, to ensure that older people have the possibility to live independently, under safe conditions, and have an active and meaningful existence in community with others. In contrast to many other countries, the responsibility for societal support and service for older people in Sweden lies on the municipalities, not relatives or private operators [3]. Thus, the municipality is responsible for ensuring that older people receive good housing and the support and services that they need [2]. This could also include the health care provided by health care professionals. Individuals can apply to their municipality for social services and, when granted, the services are provided by either the municipality or a private company, for a limited fee. This fee is based on the individual’s financial situation, but cannot be higher than a set amount (in 2022 this amount was SEK 2170, i.e., EUR 200, per month [4]). The social administration in Sweden is not required to identify old people in need of support, but only to act upon received applications. Thus, it is up to the individual to apply and, if granted, pay for the services received. A concern related to this is that socially vulnerable groups are less likely than non-vulnerable groups to seek care in accordance with their needs [5].

For most people, aging is associated with a decreased ability to perform daily tasks associated with an independent life, commonly referred to as activities of daily living (ADL). People are likely to need assistance with instrumental ADL (I-ADL; cooking, shopping, cleaning, and doing the laundry) before they need assistance with personal ADL (P-ADL; eating, drinking, dressing, personal hygiene, going to the toilet). The services provided according to the Social Services Act [2] are tailored to each person’s needs. Services related to I-ADL may concern safety alarms or the delivery of food (meals-on-wheels) and groceries, i.e., services that do not require personal support and aid in the care recipient’s home. Technical aids, such as mobility devices (e.g., canes and walkers), raised toilet seats etc., are important for independent living in one’s own home, but are regulated within the health care sector and not included in social services. Other services included in the Social Services Act, mainly those related to P-ADL, consist of more personal support, which requires human interaction between the caregiver and care recipient in the latter’s home.

There are two overall categories of social services that an older person can apply for: “Home care service” and “Institutional long-term care” (ILTC). Home care services constitute services within the person’s own home. ILTC implies accommodation at a nursing home and is only granted for people who need constant care due to physical, cognitive, psychological, or social needs [2].

Changes to the Swedish welfare system have occurred during the last decades [3,6], in that Sweden’s aging-in-place policy has made a sharp, unofficial, shift from formal care to more informal and family-oriented caregiving, relying on family and relatives taking on greater responsibilities for older relative’s need for service and support. In line with this, the chances of being granted social services have decreased, even amongst the oldest [7]. In particular, ILTC is only granted to individuals who are incapable of living on their own, with the highest amount of home care possible. Thus, the proportion of older people who receive formal social services has decreased.

People who do not receive social services are more frequently hospitalized than those who have social services [8]. This highlights the importance of maintaining a physically and mentally healthy and rich life for the individual, as well as for the demands on societies to provide support for an older population. These demands entail the costs and services needed to ensure a proper quality of life for older people. Efficient strategies for such services and support can differ, depending on the preferences of the aging population, gender issues, as well as local variations in policies and budgets among municipalities [9]. However, the consequences of such differences are to a large extent unknown.

We selected four factors to assess potential sociodemographic impacts on use of social services. Sex and living alone were chosen, as previous studies have shown [10,11,12] health-seeking differences and use of home care hours for both. Place of birth was selected, to address concerns about difficulties for immigrants in seeking social service based on language difficulties, cultural differences in health-seeking behavior, and lack of societal inclusion [13,14]. Finally, type of housing, in terms of living in an apartment or in a single-family house, was included, as this might be an indication of their vulnerability, in terms of being healthy enough to take care of a house and be living on their own.

Thus, the aims of this study were to describe the use of different forms of social services and granted ILTC in an older population in Sweden and to evaluate the impact of sex, place of birth, cohabitation status, and type of housing for one decade (2010–2019). This was done in in two sociodemographically different municipalities in southern Sweden, to establish if there were general differences in terms of the provided social services that might serve as fundament for future efforts and distribution of social care among older people.

## 2. Materials and Methods

### 2.1. Study Design

This was a cross-sectional register-based study on people aged 65 years or older living in Malmö and Kristianstad, two municipalities in the most southern part of Sweden (Scania), during 2010, 2015, and 2019. As described in more detail below, register data were used to identify the study population, as well as to collect information on sociodemographic factors and social services, ILTC, and mobility devices.

### 2.2. Study Context

Measured by number of inhabitants, Malmö is the third largest municipality in Sweden, with a population ranging from around 299,000 to 344,000 during the study years [15]. However, geographically, Malmö is one of the smaller municipalities in Sweden, covering an area of around 330 square kilometers. Thus, it is one of the most densely populated municipalities in Sweden (2193 inhabitants/km^2^). In contrast, Kristianstad is the 25th largest municipality in Sweden with a population, ranging from around 79,500 to 85,700 during the study years, and covers an area of 1 800 square kilometers (69 inhabitants/km^2^) [15]. The vast difference in population size and density between the two municipalities contributes to disparities in sociodemographic conditions, which may impact need and provision of care and social service, as well as the ability of the municipalities to provide social services.

### 2.3. Data Sources

This study was based on three national registers. The register of the total population (RTB) is maintained by Statistics Sweden, the agency responsible for official statistics and other government statistics. It comprises demographic data on all people residing in Sweden. Through this, we identified all people aged 65 years and more and living in Malmö or Kristianstad on 31 December, 2010, 2015, or 2019. Furthermore, we collected information on cohabitation status and type of housing for the 31 December, the year prior to inclusion (i.e., 2009, 2014, and 2018, respectively). The social services register, maintained by the Swedish National Board of Health and Welfare, comprises data on all social services, including ILTC, provided according to the Social Services Act [2]. From this register, we obtained data on social services for each study year (i.e., 2010, 2015, and 2019). Technical aids supplied by the municipality are registered in the Sesam register using ISO (International Organization for Standardization) codes, together with the date when the person received the aid and, when relevant, the date when the aid was returned. For this study, we retrieved information on all mobility aids (ISO codes 120303, 120306, 120309, 120312, 120316, 120603, 120606, 120609, 120612, 121806, 122203, 122218, 122306, and 122409) provided to all people in Malmö and Kristianstad between 1 January 2010 and 31 December 2019.

### 2.4. Sociodemographic Factors

Social services, ILTC, and mobility devices were assessed for four sociodemographic factors:

1. Sex (woman or man).
2. Place of birth (born in Sweden or abroad).
3. Cohabitant status (living alone or cohabiting) on the 31st of December the year before the study year. This term focuses on the living condition and not on the civil status of an individual.
4. Type of housing (living in an apartment or single-family house (house)) on the 31st of December the year before the study year.

### 2.5. Social Services, ILTC, and Mobility Devices

Use of social services and mobility devices were aggregated into a four-level-outcome, based on the level of assistance provided at each level:

1. ILTC: People who were registered as living in or moving to an institutional long-term care home during the year.
2. Major assistance: People who had home care service, in the form of personal care, respite service, or companion service; hence, individual’s dependent on personal assistance to be able to continue to live in their own housing.
3. Minor assistance: People registered as having a safety alarm, meals-on-wheels, or using mobility devices, such as wheelchairs, walkers, walking frames, crutches, or canes; thus, those who only need “non-personal” aid to live an independent life in their own housing.
4. No assistance: People with no registered use of social service or mobility devices.

People were classified based on the highest level of assistance needed. Thus, individuals who were granted accommodation in an ILTC home during a study year belonged to this group, and this group only, although they might also be registered for minor assistance in terms of e.g., mobility aid or safety alarm.

In 2010, only yearly data (i.e., one data point for the whole year) were available, and thus only one registered mobility device or service record during the year was required to be included in any of the four groups. In 2015 and 2019, monthly data were available. For these years, to be considered as having major assistance, at least three months of the relevant social services during the year, or the relevant social services during both November and December of each respective year, were required. This restriction was made to avoid including people that might only have had a temporary need of major services, e.g., post-surgery or after being hospitalized.

### 2.6. Study Populations

Six cohorts were established, comprising people living in Malmö or Kristianstad, aged 65 years or more, and alive 31 December, 2010, 2015, and 2019.

As we were unable to determine the cohabitation status and type of housing for people who moved from other municipalities to Malmö or Kristianstad in 2010, 2015, and 2019, or who had missing data on residence on those dates, these were excluded (n = 604, n = 790, and n = 782 for 2010, 2015, and 2019, respectively). We further excluded people with a need for supported living during any time during the study period (n = 95 in 2010, n = 128 in 2015, and n = 123 in 2019), or in housing (taxation unit) other than a house or apartment, or with missing data on type of housing (n = 1101 in 2010, n = 1389 in 2015, and n = 1432 in 2019). Using information from the RTB, we categorized people as cohabiting if their family type was married/partnership (with or without children) or cohabiting (with or without children). People listed as living in a single parent or single household were categorized as living alone. However, we excluded those with a role in the family listed as “child”, as the living situation and need for these might go beyond the scope of this study (n = 26 in 2010, n = 40 in 2015, and n = 53 in 2019).

Thus, the final cohorts, i.e., those used in the analyses, for Malmö comprised 45,195, 47,892, and 52,552 people for 2010, 2015, and 2019, respectively. The corresponding numbers for Kristianstad were 15,237, 16,971, and 18,046. The age distribution for the sociodemographic groups in the six cohorts were similar across years and municipalities (Table 1).

There were distinct differences in sociodemographic factors between the two municipalities cohorts (Table 2). This was especially apparent when it came to inhabitants born abroad, where the number of immigrants in Malmö was 22–28%, while the corresponding numbers for Kristianstad were 7–9% (Table 2). In addition, there was a clear difference in cohabitant status, where the majority of people in Malmö lived alone (≈54%), while in Kristianstad, most people were cohabiting (≈57%). The same pattern was found for housing situation, where a noticeable number in Malmö lived in apartments (≈74%), while most in Kristianstad lived in houses (≈67%).

### 2.7. Statistics

To address the study objectives, inferential statistics was applied. The associations between sociodemographic factors and level of support (none, minor, major, and ILTC) were assessed using the relative risk (RR) of receiving such support for men vs. women, born in Sweden vs. born abroad, living alone vs. cohabiting, and living in apartment vs. living in single-family house. RRs with 95% confidence intervals (CIs) were estimated using generalized linear models, with Poisson distribution and identity link. Separate analyses were performed for minor assistance vs. no assistance, major assistance vs. no assistance, and ILTC vs. no assistance. For each sociodemographic factor, RRs were estimated in models including only the factor itself (crude), models including the factor itself and year of birth as a continuous variable (adjusted), and in models including all sociodemographic factors and year of birth (multivariate). For each pair of sociodemographic factors, the interaction was evaluated in models including both factors and their cross-term, as well as year of birth.

## 3. Results

The results are presented below by sociodemographic factor.

### 3.1. Level of Assistance Provided According to Sex

Women were consistently more likely than men to receive minor or major assistance and ILTC, although statistical significance was not always reached (Table 3). In Kristianstad in 2019, the increase in major assistance for women was higher among those living alone than among those cohabiting; and in Malmö in 2010, the increase in ILTC was higher among those born in Sweden (Table A1). No other interaction was found between sex and the other sociodemographic factors.

### 3.2. Level of Assistance Provided According to Place of Birth

People born in Sweden were consistently more likely than those born abroad to receive major assistance. There were indications of the same pattern for minor assistance and ILTC (Table 4). Stratified analyses revealed that an increased likelihood of major assistance and ILTC was primarily found among those living alone (Table A2). No other consistent interactions were found between place of birth and the other sociodemographic variables.

### 3.3. Level of Assistance Provided According to Cohabitant Status

Living alone was consistently a risk factor for all three outcomes (minor and major assistance, and ILTC) across years and municipalities (Table 5). Stratified analyses showed that the increase in major assistance and ILTC associated with living alone was higher among those living in apartments than those living in houses in 2010 and 2015 (Table A3). Moreover, in Malmö, those being born in Sweden and living alone were more likely to be provided with major assistance and ILTC than those being born abroad and living alone. No other consistent interactions were found between cohabitation and the other sociodemographic variables.

### 3.4. Level of Assistance Provided According to Type of Housing

Those living in an apartment rather than a single-family house were consistently more likely to have all three outcomes (minor and major assistance, and ILTC) across years and municipalities, although this was not always statically significant (Table 6). Stratified analyses revealed that the increase in major assistance and ILTC associated with living in an apartment was higher among those living alone than those cohabiting (Table A4).

## 4. Discussion

Being a woman and living alone were associated with a higher use of both minor and major assistance and living in an apartment (as opposed to a house) was associated with a higher use of major assistance. Although being born in Sweden did not affect the use of social services in itself, living alone carried a higher risk among those born in Sweden than among those born abroad.

The focus of this study was older people in different sociodemographic groups and their use of social services. In the interpretation of the results, it is important to remember that we have measured use of social services, not need of them. Thus, discrepancies between groups (e.g., those between men and women) are not necessarily due to actual differences in need, but may also come from people with need finding assistance elsewhere (e.g., from an informal caregiver, such as a relative, partner or friend), health-seeking behavior or experiencing barriers to applying for social services (e.g., due to lack of knowledge about the possibility of obtaining support or how to apply for it).

In the present study, women were more likely than men to receive both minor and major assistance. This is in line with a previous study by Brändström et al. [10], in which Swedish women received twice as much home care service as men. One possible explanation could be differences in health-seeking behavior between men and women, as women tend to seek more health care in response to both physical and mental health concerns [12]. Women in Sweden, as well as in the rest of the EU, tend to live longer than men [1,16]. One consequence of this is that more women live alone at an advanced age (75% at the end of their lives compared to 30% among men [16]). However, in our study the differences between men and women in use of social services remained, even after adjusting for age, as well as place of birth, cohabitant status. This indicates that there may be other aspects involved. This is also supported by Mackian [11], who states that health-seeking behavior is not an isolated event but part of a major life puzzle involving an individual’s social, cultural, and socioeconomic situation.

Among those living alone, people born in Sweden were more likely to receive major assistance than those born abroad. This may be due to differences in health care seeking behavior and reliance on family bonds for support and help among people born in Sweden and those born abroad [13]. This possible explanation is supported by a study that estimated that, compared to Spain [17], older persons in Sweden provided almost double the amount of caregiving hours. These differences are mostly due to differences in demography and household structures, as the large majority of older people in Sweden tend to live with one partner only or alone, whereas a more complex family structure is predominant in Spain [17]. Another possible explanation for the discrepancy between those born in Sweden and those born abroad is that services for older people are not as accessible to older migrants, because of an inadequate provision of information and a lack of policy integration [14]. Even so, a study by Songur [18] revealed that in Sweden, immigrants from the middle-East and Africa used as much social services as Swedes, but refrained from using ILTC by compensating for these increased needs with family or informal caregivers. The older immigrants’ use of social services was also enabled by younger family members taking care of applications and communication between the care recipient and the social administration [18]. That this growing group of older migrants might be discriminated against in obtaining formal care and must rely upon informal caregivers is of considerable societal concern.

In the present study, people living in apartments were more likely than those living in single-family houses to receive major assistance. One explanation could be that people in need of increased help and support might have difficulties taking care of a house and garden and thus might have already moved into an apartment at an earlier stage [19]. This hypothesis is strengthened by a previous study, which showed that among older Swedish house owners it was more common to change from a house to an apartment with an increase in age [19]. This study also revealed that a change of marital status, such as becoming a widow/widower or divorced, increased the probability of moving. Such changes in life situation would also mean that an informal caregiver, in terms of a spouse, would disappear. Our findings support this by showing an interaction between living alone and living in an apartment, in terms of a higher relative risk for major assistance for those living alone in an apartment than those living alone in a single-family house (and, correspondingly, for those living alone in an apartment and those cohabiting in an apartment).

The results from the present study indicate that older people receiving social services are primarily those living alone, which is in line with other studies [6,16]. This implies that for those living together with a partner or other cohabitant, the partner/cohabitant is the one who provides, e.g., personal help and other household chores such as washing and cleaning [16]. These informal caregivers are most probably older partners fulfilling the needs that otherwise would have been provided for by formal care. This is supported by a study by Jegermalm and Torgé [20], in which the cohabitant family carer was most likely married and 50% provided care for a spouse (often a person older than 75 years). This is worth careful consideration, as informal caregivers to older people, in case of a partner or cohabitant, will most likely be of an older age themselves and might thus have limited health and physical strength. However, even when the informal caregiver is someone other than a partner, e.g., a child, their age, health, and physical strength may be an issue. Since an older population increases the age of the parents, many adult children have to take responsibility for their parents, even when they themselves are of advanced age and might need support and health care. This strain, also in the sense of time and possible economic cutbacks, that the informal caregiver burden might imply upon individuals should not be neglected. A study by Berglund et al. [5] revealed that informal caregivers were more likely than non-caregivers to report non-adherence to treatment, and to report refraining from seeking medical care for their own care needs.

In most cases, when there is a close non-cohabiting relative acting as an informal caregiver, this is often a woman [16,21]. As women in the past decades have begun to work full-time outside the home, their roles as informal caregivers is of concern, as they often, still, have the major responsibility for the home and care of children. Thus, the addition of being an informal caregiver puts a major strain on them. These high informal workloads often result in a trade-off, where the women need to choose between cutting back on workhours or accessing supplemental help. A trade-off that is not so much based on the care recipient’s (i.e., the older relative’s) well-being, as that of the caregiver’s [9]. A study by Stanfors et al. [21] on caregiving times and trade-offs showed that in Sweden, in contrast to the United Kingdom and Canada, caregivers did not trade off time in paid work for caregiving, but instead they had less leisure time. They argue that trade-offs with leisure should not be ignored but demanded further investigation because they likely have health implications, especially for women.

Despite the notable difference between the two municipalities in cohabitant status and types of housing, the patterns of obtained social service for different sociodemographic groups were the same in the two municipalities, where women and individuals living alone seem to receive more assistance. These findings also remained consistent across all three study years. This consistency strengthens our findings and stresses the fact that there are sociodemographic differences in the use of social services and ILTC in the older Swedish population.

A strength of the present study is that municipalities are required to report all care and services provided according to the Swedish Social Services Act to the National Board of Health and Welfare. Thus, the coverage of data is high. However, the level of detail and classification of services and their delivery differed between 2010 and the following years (2015 and 2019). However, as our major findings remained stable throughout all years and both municipalities, we believe this would have had negligible effects on our results.

Another study limitation is that we were unable to identify couples in this study. Although this does not affect the results in the present study, it prevents validation and quantification of the extent and burden the role of informal caregiver might impose on spouses.

There seems to be a high risk of discrimination against certain groups of older people potentially in need of social services, as well as against informal caregivers. Women and immigrants were mentioned earlier, but previous studies have also shown that individuals with higher income and education are more likely to pay for extra hours of services, both for themselves and for their relatives [10]. These groups are also more likely to be able to communicate with the authorities and thus increase the chances of claiming their legal rights to formal care [6,10,16,22]. The use and need of informal caregivers in Swedish society to obtain support and care is problematic. A study by Alwin et al. [21] concluded that the care provided by informal caregivers for frail older people is substantial and represents a considerable economic value. Although their calculations were associated with some uncertainty, the population size indicated that supporting informal caregivers should be a prime concern for society [21].

From our results, it is obvious that discrepancies either in need of or provision of social services for different socio-economic groups exist. Furthermore, these discrepancies are independent of municipality differences in sociodemographic structure and organization of social services. Moreover, they remained stable during the investigated decade. As the study was performed in a Swedish setting, the results are not necessarily generalizable to other countries, with other forms of care of older people. However, it could be argued that if discrepancies regarding provision of such care are found in Sweden, where the welfare system is designed to provide equal support for all, regardless of socio-economic ability, in countries with policies that rely more heavily on the individual’s ability to provide for themselves and their families, sociodemographically vulnerable groups may face major difficulties during ageing.

There is a need to avoid discriminating against older people, in terms of possibilities to live a healthy and independent life or becoming a major burden for potential informal caregivers (in terms of an increased workload and thus cutbacks in both time, health, and economic factors). Our study highlights the need for further research on the need and provision of social services, and efforts of informal caregivers, within different sociodemographic groups.

## 5. Conclusions

Older people’s use of social services seems to be subject to their sociodemographic characteristics. It is therefore likely that there are varying discrepancies between individual needs and the provision of social services (demanding personal assistance) among sociodemographic groups in the older population in Sweden. When society does not meet the needs of the older individual, this might be compensated for by informal caregivers. There is a major risk that supporting older relatives and partners/cohabitants poses an extra burden, and thus affects certain groups negatively. Older partners or cohabitants, children to individuals of advanced age, women, and people of another ethnicity might be overrepresented as informal caregivers and thus could have a substantial extra workload, limiting or decreasing their leisure time, physical and psychological health, and income.

Future research should focus on targeted studies of how needs and services are met, or compensated for, among older individuals in different socioeconomic groups and their cohabitants, relatives, and other possible informal caregivers. It is particularly important to assess these differences in countries with varying policies for the care of older people.

## Figures and Tables

**Table 1:** Age distribution (n, minimum (min), maximum (max), quartiles (Q1 and Q3), mean and median) for sociodemographic factors (sex, place of birth, cohabitant status, and type of housing) in the six cohorts for people 65 years and older, living in Malmö and Kristianstad in 2010, 2015, and 2019.

		Malmö					Kristianstad				
		n	Min–Max	Q1–Q3	Mean	Median	n	Min–Max	Q1–Q3	Mean	Median
**2010**	Total	45,195	65–103	69–82	76	75	15,237	65–104	68–81	75	74
Sex	Men	18,747	65–101	68–80	75	73	6890	65–101	68–79	74	72
	Women	26,448	65–103	70–83	77	76	8347	65–104	69–82	76	75
Place of birth	Sweden	35,402	65–103	69–82	76	75	14,185	65–104	68–81	75	74
	Abroad	9793	65–101	68–79	74	73	1052	65–95	69–78	74	72
Cohabitant status	Living alone	24,714	65–103	70–84	77	76	6611	65–104	70–84	77	77
	Cohabiting	20,481	65–101	68–79	74	73	8626	65–101	68–78	73	72
Type of housing	Apartment	33,757	65–103	69–83	76	75	5204	65–104	70–83	77	77
	House	11,438	65–100	68–79	74	73	10,033	65–104	68–79	74	73
**2015**	Total	47,892	65–104	69–81	75	74	16,971	65–103	69–80	75	73
Sex	Men	20,725	65–103	68–79	74	73	7874	65–103	69–79	74	73
	Women	27,167	65–104	69–82	76	75	9097	65–103	69–81	75	74
Place of birth	Sweden	35,830	65–104	69–81	76	74	15,667	65–103	69–80	75	73
	Abroad	12,062	65–104	68–79	74	72	1304	65–97	68–78	74	73
Cohabitant status	Living alone	25,748	65–104	69–83	77	75	7173	65–103	70–83	77	76
	Cohabiting	22,144	65–99	68–78	74	72	9798	65–99	68–78	73	72
Type of housing	Apartment	35,314	65–104	69–81	76	74	5420	65–102	70–83	77	76
	House	12,578	65–102	68–79	74	73	11,551	65–103	68–78	74	72
**2019**	Total	50,329	65–107	69–80	75	74	18,046	65–106	69–80	75	74
Sex	Men	22,273	65–105	69–79	74	73	8494	65–102	69–79	75	74
	Women	28,056	65–107	69–81	76	74	9552	65–106	70–81	76	74
Place of birth	Sweden	36,020	65–105	69–81	76	74	16,410	65–106	70–80	75	74
	Abroad	14,309	65–107	68–78	74	72	1636	65–101	68–78	74	72
Cohabitant status	Living alone	26,930	65–107	69–82	76	75	7698	65–106	70–83	77	76
	Cohabiting	23,399	65–103	68–78	74	73	10,348	65–98	69–78	74	73
Type of housing	Apartment	37,250	65–107	69–80	75	74	5632	65–104	70–82	77	76
	House	13,079	65–102	69–79	74	73	12,414	65–106	69–79	75	73

**Table 2:** Distribution of the four sociodemographic factors (sex, place of birth, cohabitation, and type of housing) for people 65 years and older, living in Malmö and Kristianstad in 2010, 2015, and 2019.

		Malmö	Kristianstad
2010		n (%)	n (%)
Sex	Men	18,747 (41)	6890 (45)
	Women	26,448 (59)	8347 (55)
Place of birth	Sweden	35,402 (78)	14,185 (93)
	Abroad	9793 (22)	1052 (7)
Cohabitant status	Living alone	24,714 (55)	6611 (43)
	Cohabiting	20,481 (45)	8626 (57)
Type of housing	Apartment	33,757 (75)	5204 (34)
	House	11,438 (25)	10,033 (66)
**2015**			
Sex	Men	20,725 (43)	7874 (46)
	Women	27,167 (57)	9097 (54)
Place of birth	Sweden	35,830 (75)	15,667 (92)
	Abroad	12,062 (25)	1304 (8)
Cohabitant status	Living alone	25,748 (54)	7173 (42)
	Cohabiting	22,144 (46)	9798 (58)
Type of housing	Apartment	35,314 (74)	5420 (32)
	House	12,578 (26)	11,551 (68)
**2019**			
Sex	Men	22,273 (44)	8494 (47)
	Women	28,056 (56)	9552 (53)
Place of birth	Sweden	36,020 (72)	16,410 (91)
	Abroad	14,309 (28)	1636 (9)
Cohabitant status	Living alone	26,930 (54)	7698 (43)
	Cohabiting	23,399 (46)	10,348 (57)
Type of housing	Apartment	37,250 (74)	5632 (31)
	House	13,079 (26)	12,414 (69)

**Table 3:** Number of men and women, stratified by year and municipality, in the different outcome categories (n (%)), and relative risks with 95% confidence intervals for comparisons of women vs. men, adjusted for year of birth and the other sociodemographic variables (place of birth, cohabitant status, type of housing). Bold indicates statistically significant differences.

	Malmö			Kristianstad		
2010	Women	Men	Women vs. Men	Women	Men	Women vs. Men
No assistance	19,182 (73)	15,823 (84)	Ref	6367 (76)	5993 (87)	Ref
Minor assistance	2406 (9)	1054 (6)	**1.035 (1.015–1.056)**	794 (10)	379 (6)	1.030 (0.996–1.065)
Major assistance	3805 (14)	1461 (8)	**1.080 (1.061–1.100)**	790 (9)	352 (5)	**1.053 (1.020–1.088)**
ILTC ^1^	1055 (4)	409 (2)	**1.043 (1.022–1.064)**	396 (5)	166 (2)	**1.044 (1.010–1.079)**
**2015**						
No assistance	18,815 (69)	16,938 (82)	Ref	6570 (72)	6530 (83)	Ref
Minor assistance	3741 (14)	1715 (8)	**1.055 (1.035–1.074)**	1223 (13)	668 (8)	**1.041 (1.010–1.074)**
Major assistance	3808 (14)	1737 (8)	**1.075 (1.056–1.094)**	915 (10)	470 (6)	**1.057 (1.026–1.090)**
ILTC ^1^	803 (3)	335 (2)	**1.041 (1.020–1.062)**	389 (4)	206 (3)	**1.039 (1.006–1.073)**
**2019**						
No assistance	19,714 (70)	18,160 (82)	Ref	6873 (72)	6971 (82)	Ref
Minor assistance	2551 (9)	1290 (6)	**1.040 (1.021–1.060)**	756 (8)	480 (6)	1.027 (0.995–1.059)
Major assistance	5387 (19)	2611 (12)	**1.088 (1.070–1.106)**	1605 (17)	868 (10)	**1.081 (1.052–1.111)**
ILTC ^1^	404 (1)	212 (1)	1.015 (0.995–1.036)	318 (3)	175 (2)	**1.035 (1.003–1.068)**

^1^ Institutional long-term care.

**Table 4:** Number of people born in Sweden and abroad, stratified by year and municipality, in the different outcome categories (n (%)), and relative risks with 95% confidence intervals for comparisons of being born in Sweden vs. being born abroad, adjusted for year of birth and the other sociodemographic variables (sex, cohabitant status, type of housing). Bold indicates statistically significant differences.

	Malmö			Kristianstad		
2010	Sweden	Abroad	Sweden vs. Abroad	Sweden	Abroad	Sweden vs. Abroad
No assistance	26,839 (76)	8166 (83)	Ref	11,454 (81)	906 (86)	Ref
Minor assistance	2836 (8)	624 (6)	**1.027 (1.003–1.051)**	1113 (8)	60 (6)	1.037 (0.973–1.106)
Major assistance	4481 (13)	785 (8)	**1.110 (1.086–1.134)**	1078 (8)	64 (6)	**1.065 (1.001–1.132)**
ILTC ^1^	1246 (4)	218 (2)	**1.063 (1.038–1.088)**	540 (4)	22 (2)	**1.094 (1.026–1.167)**
**2015**						
No assistance	26,304 (73)	9449 (78)	Ref	12,071 (77)	1029 (79)	Ref
Minor assistance	4128 (12)	1328 (11)	1.016 (0.995–1.038)	1748 (11)	143 (11)	1.024 (0.968–1.084)
Major assistance	4474 (12)	1071 (9)	**1.082 (1.060–1.104)**	1292 (8)	93 (7)	**1.071 (1.011–1.133)**
ILTC ^1^	924 (3)	214 (2)	**1.039 (1.016–1.063)**	556 (4)	39 (3)	1.062 (1.000–1.127)
**2019**						
No assistance	26,687 (74)	11,187 (78)	Ref	12,543 (76)	1301 (80)	Ref
Minor assistance	2503 (7)	1338 (9)	0.985 (0.065–1.005)	1120 (7)	116 (7)	1.016 (0.963–1.072)
Major assistance	6370 (18)	1628 (11)	**1.110 (1.090–1.130)**	2299 (14)	174 (11)	**1.106 (1.053–1.161)**
ILTC ^1^	460 (1)	156 (1)	1.012 (0.990–1.034)	448 (3)	45 (3)	1.033 (0.979–1.091)

^1^ Institutional long-term care.

**Table 5:** Number of people living alone and cohabitating, stratified by year and municipality, in the different outcome categories (n (%)), and relative risks with 95% confidence intervals for comparisons of living alone vs. cohabiting, adjusted for year of birth and the other sociodemographic variables (sex, place of birth, type of housing). Bold indicates statistically significant differences.

	Malmö			Kristianstad		
2010	Living Alone	Cohabitant	Alone vs. Cohabitant	Living Alone	Cohabitant	Alone vs. Cohabitant
No assistance	17,052 (69)	17,953 (88)	Ref	4547 (69)	7813 (91)	Ref
Minor assistance	2268 (9)	1192 (6)	**1.039 (1.018–1.061)**	777 (12)	396 (5)	**1.078 (1.041–1.116)**
Major assistance	4190 (17)	1076 (5)	**1.203 (1.180–1.226)**	840 (13)	302 (4)	**1.181 (1.143–1.221)**
ILTC ^1^	1204 (5)	260 (1)	**1.125 (1.102–1.148)**	447 (7)	115 (1)	**1.171 (1.131–1.212)**
**2015**						
No assistance	16,949 (66)	18,804 (85)	Ref	4642 (65)	8458 (86)	Ref
Minor assistance	3502 (14)	1954 (9)	**1.050 (1.030–1.070)**	1108 (15)	783 (8)	**1.077 (1.043–1.112)**
Major assistance	4394 (17)	1151 (5)	**1.225 (1.202–1.247)**	983 (14)	402 (4)	**1.190 (1.153–1.228)**
ILTC ^1^	903 (4)	235 (1)	**1.094 (1.072–1.117)**	440 (6)	155 (2)	**1.158 (1.120–1.198)**
**2019**						
No assistance	18,038 (67)	19,836 (85)	Ref	5037 (65)	8807 (85)	Ref
Minor assistance	2265 (8)	1576 (7)	**1.021 (1.001–1.041)**	594 (8)	642 (6)	1.030 (0.987–1.054)
Major assistance	6139 (23)	1859 (8)	**1.247 (1.226–1.268)**	1700 (22)	773 (7)	**1.241 (1.206–1.277)**
ILTC ^1^	488 (2)	128 (1)	**1.053 (1.032–1.075)**	367 (5)	126 (1)	**1.128 (1.091–1.166)**

^1^ Institutional long-term care.

**Table 6:** Number of people living in a house or apartment, stratified by year and municipality, in the different outcome categories (n (%)), and relative risks with 95% confidence intervals for comparisons of living in an apartment vs. living in a house, adjusted for year of birth and the other sociodemographic variables (sex, place of birth, cohabitant status). Bold indicates statistically significant differences.

	Malmö			Kristianstad		
2010	House	Apartment	Apartment vs. House	House	Apartment	Apartment vs. House
No assistance	9954 (87)	25,051 (74)	Ref	8663 (86)	3697 (71)	Ref
Minor assistance	680 (6)	2780 (8)	1.021 (0.998–1.044)	619 (6)	554 (11)	1.034 (0.997–1.072)
Major assistance	637 (6)	4629 (14)	**1.102 (1.078–1.126)**	534 (5)	608 (12)	**1.087 (1.050–1.125)**
ILTC ^1^	167 (1)	1297 (4)	**1.055 (1.031–1.080)**	217 (2)	345 (7)	**1.113 (1.074–1.154)**
**2015**						
No assistance	10 565 (84)	25,188 (71)	Ref	9565 (83)	3535 (65)	Ref
Minor assistance	1019 (8)	4437 (13)	**1.039 (1.017–1.061)**	1053 (9)	838 (15)	**1.057 (1.021–1.095)**
Major assistance	824 (7)	4721 (13)	**1.078 (1.056–1.101)**	664 (6)	721 (13)	**1.122 (1.085–1.160)**
ILTC ^1^	170 (1)	968 (3)	**1.033 (1.009–1.057)**	269 (2)	326 (6)	**1.109 (1.070–1.085)**
**2019**						
No assistance	10,864 (83)	27,010 (73)	Ref	10,149 (82)	3695 (66)	Ref
Minor assistance	718 (5)	3123 (8)	**1.028 (1.006–1.050)**	717 (6)	519 (9)	**1.047 (1.010–1.085)**
Major assistance	1397 (11)	6601 (18)	**1.064 (1.043–1.084)**	1313 (11)	1160 (21)	**1.125 (1.091–1.159)**
ILTC ^1^	100 (1)	516 (1)	1.013 (0.990–1.036)	235 (2)	258 (5)	**1.079 (1.041–1.119)**

^1^ Institutional long-term care.

## Data Availability

Since the data comprise information about single individuals and are detailed enough to enable identification of, at least, some of the people included, they cannot be made publicly available. However, as the database was compiled with national register data, other researchers may contact the register holders (the Swedish National Board of Health and Welfare, Statistics Sweden, and Malmö and Kristianstad municipalities) to obtain access to the registries used in this study, and thereby generate an identical database.

## Appendix A

**Table A1:** RR for women vs. men with *p*-values for interaction.

			Minor vs. None				Major vs. None				ILTC vs. None			
			RR	95% CI		*p*	RR	95% CI		*p*	RR	95% CI		*p*
Malmö	2010	Crude	1.046	1.026	1.067		1.139	1.119	1.159		1.075	1.054	1.096	
		Adjusted	1.034	1.014	1.054		1.079	1.060	1.098		1.047	1.027	1.068	
		Multivariate	1.027	1.007	1.048		1.043	1.024	1.062		1.025	1.004	1.045	
		Cohabiting: Cohabiting	1.013	0.985	1.041	0.193	1.037	1.009	1.065	0.919	1.016	0.987	1.045	0.960
		Cohabiting: Living alone	1.037	1.008	1.068		1.031	1.005	1.058		1.007	0.979	1.037	
		Housing: Apartment	1.034	1.011	1.058	0.714	1.075	1.053	1.098	0.185	1.044	1.021	1.069	0.375
		Housing: House	1.027	0.990	1.066		1.053	1.016	1.092		1.030	0.991	1.069	
		Place of birth: Abroad	1.035	0.993	1.078	0.867	1.073	1.032	1.116	0.296	1.019	0.977	1.062	0.044
		Place of birth: Sweden	1.034	1.012	1.057		1.081	1.060	1.103		1.057	1.033	1.080	
	2015	Crude	1.068	1.048	1.087		1.127	1.108	1.147		1.061	1.040	1.082	
		Adjusted	1.055	1.036	1.074		1.073	1.054	1.092		1.046	1.026	1.067	
		Multivariate	1.046	1.027	1.066		1.040	1.021	1.058		1.032	1.012	1.053	
		Cohabiting: Cohabiting	1.027	1.000	1.054	0.064	1.028	1.002	1.056	0.703	1.018	0.990	1.047	0.736
		Cohabiting: Living alone	1.063	1.034	1.092		1.032	1.007	1.058		1.021	0.992	1.051	
		Housing: Apartment	1.057	1.035	1.080	0.333	1.072	1.050	1.094	0.115	1.046	1.022	1.071	0.443
		Housing: House	1.037	1.002	1.074		1.043	1.008	1.080		1.031	0.993	1.070	
		Place of birth: Abroad	1.075	1.037	1.114	0.270	1.080	1.042	1.118	0.974	1.035	0.996	1.076	0.429
		Place of birth: Sweden	1.048	1.026	1.071		1.070	1.049	1.092		1.049	1.026	1.074	
	2019	Crude	1.045	1.026	1.065		1.142	1.124	1.160		1.025	1.005	1.045	
		Adjusted	1.043	1.024	1.062		1.084	1.066	1.101		1.023	1.003	1.043	
		Multivariate	1.039	1.020	1.059		1.050	1.033	1.067		1.015	0.995	1.035	
		Cohabiting: Cohabiting	1.025	0.998	1.052	0.156	1.031	1.006	1.057	0.170	1.004	0.977	1.032	0.721
		Cohabiting: Living alone	1.053	1.025	1.082		1.054	1.031	1.077		1.012	0.983	1.041	
		Housing: Apartment	1.044	1.022	1.067	0.534	1.082	1.062	1.102	0.334	1.024	1.000	1.048	0.657
		Housing: House	1.031	0.995	1.068		1.065	1.031	1.099		1.013	0.977	1.052	
		Place of birth: Abroad	1.070	1.035	1.107	0.067	1.096	1.062	1.131	0.625	1.026	0.990	1.064	0.807
		Place of birth: Sweden	1.031	1.008	1.054		1.078	1.058	1.098		1.021	0.997	1.045	
Kristianstad	2010	Crude	1.049	1.015	1.083		1.099	1.065	1.134		1.088	1.053	1.124	
		Adjusted	1.035	1.002	1.069		1.065	1.032	1.099		1.056	1.022	1.091	
		Multivariate	1.025	0.991	1.059		1.038	1.005	1.072		1.031	0.997	1.066	
		Cohabiting: Cohabiting	1.014	0.972	1.058	0.519	1.015	0.973	1.058	0.173	1.013	0.970	1.058	0.472
		Cohabiting: Living alone	1.030	0.976	1.087		1.047	0.996	1.102		1.018	0.966	1.073	
		Housing: Apartment	1.048	0.988	1.112	0.473	1.075	1.017	1.136	0.296	1.051	0.992	1.114	0.324
		Housing: House	1.024	0.985	1.065		1.046	1.006	1.087		1.037	0.996	1.080	
		Place of birth: Abroad	1.058	0.934	1.197	0.765	1.079	0.957	1.217	0.803	1.054	0.929	1.194	0.898
		Place of birth: Sweden	1.033	0.999	1.069		1.064	1.030	1.099		1.056	1.021	1.092	
	2015	Crude	1.059	1.027	1.091		1.097	1.065	1.130		1.070	1.036	1.104	
		Adjusted	1.049	1.018	1.081		1.071	1.040	1.104		1.057	1.024	1.090	
		Multivariate	1.038	1.007	1.071		1.045	1.013	1.077		1.037	1.004	1.071	
		Cohabiting: Cohabiting	1.022	0.983	1.063	0.267	1.031	0.990	1.073	0.478	1.012	0.971	1.055	0.406
		Cohabiting: Living alone	1.055	1.004	1.109		1.045	0.996	1.096		1.029	0.978	1.083	
		Housing: Apartment	1.068	1.010	1.130	0.340	1.081	1.025	1.141	0.327	1.069	1.009	1.133	0.229
		Housing: House	1.035	0.998	1.073		1.052	1.014	1.091		1.036	0.997	1.076	
		Place of birth: Abroad	1.061	0.952	1.182	0.854	1.113	0.998	1.242	0.506	1.074	0.958	1.204	0.805
		Place of birth: Sweden	1.048	1.015	1.081		1.068	1.035	1.102		1.055	1.021	1.090	
	2019	Crude	1.033	1.001	1.065		1.129	1.099	1.160		1.055	1.023	1.089	
		Adjusted	1.033	1.002	1.065		1.091	1.062	1.121		1.049	1.017	1.083	
		Multivariate	1.029	0.998	1.061		1.061	1.032	1.090		1.036	1.004	1.069	
		Cohabiting: Cohabiting	1.021	0.982	1.062	0.477	1.027	0.989	1.066	0.013	1.000	0.960	1.042	0.072
		Cohabiting: Living alone	1.045	0.993	1.099		1.098	1.053	1.144		1.055	1.003	1.109	
		Housing: Apartment	1.049	0.990	1.112	0.440	1.099	1.047	1.153	0.376	1.049	0.990	1.111	0.573
		Housing: House	1.022	0.985	1.060		1.071	1.036	1.107		1.037	0.999	1.076	
		Place of birth: Abroad	1.040	0.941	1.149	0.907	1.100	1.003	1.207	0.962	1.054	0.952	1.167	0.966
		Place of birth: Sweden	1.033	1.000	1.066		1.090	1.060	1.121		1.049	1.015	1.084	

**Table A2:** RR for born in Sweden vs. born abroad with *p*-values for interaction.

			Minor vs. None				Major vs. None				ILTC vs. None			
			RR	95% CI		*p*	RR	95% CI		*p*	RR	95% CI		*p*
Malmö	2010	Crude	1.023	1.000	1.047		1.094	1.071	1.118		1.051	1.027	1.076	
		Adjusted	1.009	0.986	1.032		1.034	1.012	1.057		1.019	0.996	1.043	
		Multivariate	1.011	0.988	1.035		1.048	1.025	1.071		1.028	1.004	1.053	
		Cohabiting: Cohabiting	0.999	0.966	1.033	0.214	1.004	0.971	1.038	<0.001	1.006	0.972	1.042	0.003
		Cohabiting: Living alone	1.022	0.990	1.055		1.073	1.043	1.103		1.047	1.015	1.081	
		Housing: Apartment	1.011	0.985	1.038	0.910	1.049	1.024	1.074	0.148	1.030	1.003	1.057	0.142
		Housing: House	1.010	0.958	1.064		1.016	0.965	1.070		0.996	0.944	1.051	
		Sex: Men	1.011	0.976	1.046	0.867	1.037	1.003	1.073	0.296	1.006	0.971	1.042	0.044
		Sex: Women	1.008	0.978	1.039		1.036	1.007	1.065		1.034	1.002	1.067	
	2015	Crude	1.011	0.990	1.032		1.072	1.051	1.094		1.033	1.010	1.057	
		Adjusted	0.994	0.973	1.015		1.014	0.993	1.034		1.007	0.984	1.030	
		Multivariate	0.998	0.978	1.019		1.024	1.003	1.045		1.012	0.989	1.035	
		Cohabiting: Cohabiting	0.991	0.962	1.022	0.480	0.984	0.955	1.015	<0.001	1.000	0.968	1.033	0.028
		Cohabiting: Living alone	1.001	0.973	1.030		1.046	1.019	1.075		1.025	0.993	1.057	
		Housing: Apartment	0.999	0.976	1.022	0.930	1.030	1.007	1.053	0.131	1.016	0.990	1.042	0.253
		Housing: House	1.000	0.954	1.048		1.000	0.954	1.047		0.994	0.946	1.044	
		Sex: Men	1.010	0.979	1.042	0.270	1.024	0.994	1.056	0.974	1.006	0.973	1.039	0.429
		Sex: Women	0.980	0.953	1.008		1.003	0.977	1.031		1.005	0.975	1.037	
	2019	Crude	0.981	0.962	1.001		1.105	1.085	1.125		1.009	0.988	1.031	
		Adjusted	0.971	0.952	0.991		1.026	1.008	1.045		0.994	0.973	1.015	
		Multivariate	0.975	0.955	0.995		1.035	1.016	1.054		0.996	0.975	1.018	
		Cohabiting: Cohabiting	0.981	0.953	1.009	0.470	0.991	0.964	1.019	<0.001	0.993	0.963	1.024	0.251
		Cohabiting: Living alone	0.965	0.938	0.992		1.061	1.036	1.086		1.002	0.972	1.033	
		Housing: Apartment	0.973	0.951	0.995	0.741	1.045	1.024	1.067	0.055	0.998	0.974	1.023	0.573
		Housing: House	0.984	0.941	1.028		1.004	0.963	1.046		0.990	0.945	1.037	
		Sex: Men	0.993	0.964	1.023	0.067	1.041	1.013	1.070	0.625	1.001	0.971	1.033	0.807
		Sex: Women	0.952	0.927	0.978		1.012	0.988	1.036		0.985	0.956	1.015	
Kristianstad	2010	Crude	1.025	0.962	1.092		1.035	0.974	1.101		1.060	0.994	1.130	
		Adjusted	1.006	0.944	1.072		1.001	0.941	1.064		1.018	0.955	1.086	
		Multivariate	1.013	0.951	1.080		1.020	0.959	1.085		1.039	0.974	1.109	
		Cohabiting: Cohabiting	1.003	0.920	1.095	0.665	0.991	0.910	1.080	0.301	0.994	0.910	1.085	0.085
		Cohabiting: Living alone	1.018	0.928	1.118		1.029	0.942	1.124		1.061	0.965	1.167	
		Housing: Apartment	1.008	0.920	1.105	0.945	1.016	0.931	1.109	0.670	1.020	0.930	1.119	0.479
		Housing: House	1.010	0.924	1.105		1.005	0.921	1.097		1.018	0.929	1.115	
		Sex: Men	1.023	0.930	1.126	0.765	1.023	0.932	1.122	0.803	1.030	0.936	1.134	0.898
		Sex: Women	0.994	0.913	1.083		0.985	0.908	1.069		1.011	0.927	1.103	
	2015	Crude	1.004	0.949	1.062		1.024	0.968	1.083		1.020	0.962	1.083	
		Adjusted	0.992	0.938	1.050		1.002	0.947	1.060		1.004	0.947	1.066	
		Multivariate	1.004	0.949	1.063		1.030	0.973	1.091		1.025	0.966	1.089	
		Cohabiting: Cohabiting	0.982	0.909	1.061	0.499	0.991	0.915	1.073	0.275	0.988	0.911	1.072	0.212
		Cohabiting: Living alone	1.015	0.934	1.102		1.040	0.959	1.126		1.044	0.957	1.138	
		Housing: Apartment	1.016	0.938	1.101	0.651	1.025	0.949	1.108	0.900	1.032	0.948	1.123	0.252
		Housing: House	0.991	0.914	1.075		1.026	0.943	1.116		0.987	0.906	1.075	
		Sex: Men	1.000	0.921	1.086	0.854	1.029	0.946	1.119	0.506	1.018	0.934	1.109	0.805
		Sex: Women	0.985	0.912	1.064		0.979	0.908	1.057		0.994	0.916	1.078	
	2019	Crude	1.000	0.949	1.054		1.060	1.010	1.112		1.003	0.951	1.058	
		Adjusted	0.988	0.938	1.042		1.000	0.953	1.050		0.973	0.922	1.026	
		Multivariate	1.000	0.948	1.055		1.031	0.982	1.082		0.991	0.939	1.047	
		Cohabiting: Cohabiting	0.993	0.925	1.065	0.979	0.973	0.909	1.041	0.054	0.975	0.907	1.049	0.302
		Cohabiting: Living alone	0.988	0.913	1.070		1.054	0.985	1.129		1.002	0.925	1.085	
		Housing: Apartment	0.998	0.927	1.075	0.951	1.067	0.998	1.140	0.141	1.007	0.933	1.088	0.136
		Housing: House	1.000	0.925	1.081		0.993	0.924	1.067		0.956	0.884	1.033	
		Sex: Men	0.995	0.921	1.073	0.907	1.013	0.943	1.089	0.962	0.987	0.913	1.067	0.966
		Sex: Women	0.984	0.915	1.058		0.993	0.931	1.059		0.966	0.897	1.040	

**Table A3:** RR for living alone vs. cohabiting with *p*-values for interaction.

			Minor vs. None				Major vs. None				ILTC vs. None			
			RR	95% CI		*p*	RR	95% CI		*p*	RR	95% CI		*p*
Malmö	2010	Crude	1.052	1.032	1.072		1.253	1.231	1.275		1.149	1.127	1.171	
		Adjusted	1.034	1.014	1.054		1.158	1.137	1.179		1.102	1.081	1.124	
		Multivariate	1.026	1.005	1.047		1.133	1.111	1.155		1.090	1.067	1.113	
		Housing: Apartment	1.032	1.009	1.056	0.825	1.161	1.137	1.186	<0.001	1.106	1.080	1.132	0.012
		Housing: House	1.032	0.990	1.075		1.094	1.051	1.138		1.072	1.028	1.119	
		Place of birth: Abroad	1.016	0.975	1.059	0.214	1.093	1.051	1.137	<0.001	1.060	1.016	1.105	0.003
		Place of birth: Sweden	1.040	1.018	1.063		1.179	1.155	1.203		1.118	1.093	1.143	
		Sex: Men	1.011	0.981	1.042	0.193	1.148	1.117	1.181	0.919	1.095	1.063	1.129	0.960
		Sex: Women	1.035	1.007	1.062		1.128	1.100	1.157		1.078	1.049	1.108	
	2015	Crude	1.070	1.051	1.090		1.266	1.244	1.288		1.111	1.089	1.133	
		Adjusted	1.048	1.029	1.067		1.163	1.143	1.184		1.076	1.055	1.097	
		Multivariate	1.030	1.010	1.050		1.139	1.118	1.161		1.063	1.041	1.086	
		Housing: Apartment	1.040	1.017	1.063	0.824	1.161	1.137	1.186	0.003	1.081	1.055	1.106	0.020
		Housing: House	1.043	1.003	1.084		1.118	1.077	1.161		1.044	1.001	1.088	
		Place of birth: Abroad	1.039	1.002	1.077	0.480	1.105	1.066	1.145	<0.001	1.047	1.007	1.089	0.028
		Place of birth: Sweden	1.051	1.029	1.073		1.184	1.160	1.208		1.087	1.063	1.112	
		Sex: Men	1.017	0.988	1.046	0.064	1.153	1.122	1.184	0.703	1.065	1.034	1.097	0.736
		Sex: Women	1.048	1.022	1.075		1.137	1.109	1.165		1.054	1.025	1.083	
	2019	Crude	1.035	1.017	1.055		1.287	1.267	1.308		1.059	1.038	1.080	
		Adjusted	1.028	1.009	1.047		1.173	1.154	1.193		1.047	1.027	1.068	
		Multivariate	1.014	0.994	1.034		1.148	1.129	1.168		1.041	1.020	1.063	
		Housing: Apartment	1.021	0.999	1.044	0.835	1.164	1.142	1.187	0.262	1.048	1.024	1.073	0.353
		Housing: House	1.020	0.980	1.062		1.153	1.114	1.193		1.034	0.992	1.078	
		Place of birth: Abroad	1.037	1.003	1.073	0.470	1.115	1.080	1.151	<0.001	1.031	0.994	1.069	0.251
		Place of birth: Sweden	1.022	1.000	1.045		1.196	1.173	1.219		1.054	1.030	1.079	
		Sex: Men	1.005	0.977	1.033	0.156	1.151	1.123	1.179	0.170	1.039	1.009	1.070	0.721
		Sex: Women	1.030	1.004	1.057		1.157	1.131	1.184		1.039	1.011	1.068	
Kristianstad	2010	Crude	1.093	1.058	1.130		1.221	1.183	1.260		1.216	1.176	1.256	
		Adjusted	1.055	1.020	1.091		1.134	1.098	1.171		1.121	1.083	1.159	
		Multivariate	1.046	1.010	1.084		1.113	1.075	1.151		1.098	1.060	1.138	
		Housing: Apartment	1.065	1.005	1.130	0.472	1.160	1.097	1.227	0.054	1.165	1.099	1.235	0.001
		Housing: House	1.044	1.000	1.091		1.103	1.058	1.151		1.079	1.033	1.128	
		Place of birth: Abroad	1.035	0.912	1.174	0.665	1.061	0.939	1.198	0.301	1.018	0.896	1.156	0.085
		Place of birth: Sweden	1.057	1.021	1.094		1.140	1.102	1.179		1.130	1.091	1.170	
		Sex: Men	1.039	0.986	1.094	0.519	1.103	1.049	1.160	0.173	1.109	1.053	1.168	0.472
		Sex: Women	1.049	1.002	1.099		1.118	1.069	1.169		1.090	1.040	1.142	
	2015	Crude	1.100	1.066	1.134		1.237	1.201	1.275		1.195	1.158	1.234	
		Adjusted	1.062	1.029	1.095		1.150	1.116	1.186		1.118	1.083	1.155	
		Multivariate	1.047	1.013	1.082		1.125	1.089	1.161		1.100	1.063	1.138	
		Housing: Apartment	1.085	1.026	1.147	0.196	1.209	1.144	1.277	0.002	1.167	1.100	1.237	0.007
		sing: House	1.038	0.998	1.080		1.099	1.056	1.143		1.086	1.042	1.132	
		Place of birth: Abroad	1.032	0.925	1.152	0.499	1.099	0.984	1.228	0.275	1.053	0.938	1.183	0.212
		Place of birth: Sweden	1.064	1.030	1.100		1.156	1.119	1.193		1.125	1.087	1.163	
		Sex: Men	1.035	0.987	1.085	0.267	1.131	1.080	1.185	0.478	1.099	1.047	1.154	0.406
		Sex: Women	1.062	1.018	1.109		1.128	1.081	1.178		1.099	1.051	1.150	
	2019	Crude	1.035	1.003	1.068		1.296	1.261	1.331		1.155	1.119	1.192	
		Adjusted	1.024	0.992	1.057		1.173	1.141	1.206		1.099	1.064	1.135	
		Multivariate	1.011	0.978	1.045		1.141	1.109	1.175		1.083	1.047	1.119	
		Housing: Apartment	1.016	0.959	1.077	0.936	1.183	1.125	1.243	0.168	1.130	1.065	1.199	0.046
		Housing: House	1.015	0.975	1.056		1.139	1.100	1.180		1.076	1.034	1.121	
		Place of birth: Abroad	1.031	0.931	1.142	0.979	1.096	0.997	1.204	0.054	1.052	0.948	1.168	0.302
		Place of birth: Sweden	1.023	0.989	1.058		1.181	1.147	1.216		1.104	1.067	1.142	
		Sex: Men	1.007	0.961	1.056	0.477	1.122	1.076	1.170	0.013	1.065	1.016	1.117	0.072
		Sex: Women	1.027	0.983	1.073		1.178	1.133	1.224		1.097	1.049	1.147	

**Table A4:** RR for apartment vs. house with *p*-values for interaction.

			Minor vs. None				Major vs. None				ILTC vs. None			
			RR	95% CI		*p*	RR	95% CI		*p*	RR	95% CI		*p*
Malmö	2010	Crude	1.034	1.012	1.056		1.171	1.147	1.195		1.094	1.070	1.118	
		Adjusted	1.019	0.998	1.042		1.100	1.078	1.123		1.057	1.033	1.080	
		Multivariate	1.010	0.988	1.034		1.059	1.036	1.082		1.030	1.006	1.054	
		Cohabiting: Cohabiting	1.011	0.983	1.040	0.825	1.033	1.005	1.062	<0.001	1.021	0.992	1.051	0.012
		Cohabiting: Living alone	1.013	0.975	1.052		1.108	1.070	1.147		1.065	1.024	1.106	
		Place of birth: Abroad	1.019	0.966	1.075	0.910	1.072	1.017	1.129	0.148	1.025	0.970	1.082	0.142
		Place of birth: Sweden	1.020	0.996	1.045		1.110	1.085	1.135		1.065	1.039	1.091	
		Sex: Men	1.014	0.983	1.047	0.714	1.086	1.053	1.119	0.185	1.049	1.015	1.083	0.375
		Sex: Women	1.017	0.987	1.048		1.094	1.063	1.126		1.050	1.018	1.083	
	2015	Crude	1.057	1.035	1.079		1.149	1.127	1.172		1.061	1.038	1.084	
		Adjusted	1.044	1.023	1.066		1.099	1.078	1.121		1.045	1.022	1.068	
		Multivariate	1.031	1.009	1.054		1.056	1.034	1.078		1.024	1.001	1.048	
		Cohabiting: Cohabiting	1.033	1.005	1.060	0.824	1.035	1.007	1.063	0.003	1.010	0.982	1.039	0.020
		Cohabiting: Living alone	1.036	1.000	1.074		1.094	1.059	1.130		1.064	1.024	1.105	
		Place of birth: Abroad	1.043	0.995	1.093	0.930	1.071	1.022	1.122	0.131	1.021	0.972	1.073	0.253
		Place of birth: Sweden	1.044	1.021	1.069		1.108	1.084	1.133		1.051	1.026	1.077	
		Sex: Men	1.030	1.000	1.061	0.333	1.079	1.048	1.110	0.115	1.034	1.003	1.067	0.443
		Sex: Women	1.047	1.018	1.077		1.100	1.070	1.131		1.042	1.011	1.075	
	2019	Crude	1.039	1.018	1.061		1.134	1.114	1.155		1.028	1.006	1.051	
		Adjusted	1.036	1.015	1.058		1.097	1.077	1.117		1.025	1.003	1.048	
		Multivariate	1.026	1.004	1.048		1.053	1.033	1.073		1.011	0.988	1.034	
		Cohabiting: Cohabiting	1.029	1.002	1.056	0.835	1.043	1.017	1.070	0.262	1.006	0.978	1.034	0.353
		Cohabiting: Living alone	1.034	0.996	1.072		1.064	1.034	1.096		1.031	0.992	1.071	
		Place of birth: Abroad	1.039	0.995	1.085	0.741	1.065	1.022	1.110	0.055	1.013	0.967	1.061	0.573
		Place of birth: Sweden	1.031	1.006	1.055		1.109	1.087	1.133		1.028	1.002	1.054	
		Sex: Men	1.027	0.997	1.058	0.534	1.081	1.052	1.111	0.334	1.019	0.988	1.051	0.657
		Sex: Women	1.039	1.009	1.069		1.093	1.066	1.121		1.027	0.995	1.059	
Kristianstad	2010	Crude	1.060	1.024	1.097		1.149	1.112	1.187		1.170	1.131	1.211	
		Adjusted	1.029	0.994	1.065		1.082	1.046	1.118		1.089	1.052	1.127	
		Multivariate	1.014	0.978	1.052		1.047	1.011	1.084		1.060	1.022	1.099	
		Cohabiting: Cohabiting	1.009	0.958	1.064	0.472	1.031	0.979	1.085	0.054	1.026	0.973	1.082	0.001
		Cohabiting: Living alone	1.024	0.974	1.077		1.073	1.024	1.124		1.101	1.048	1.157	
		Place of birth: Abroad	1.025	0.907	1.159	0.945	1.056	0.938	1.189	0.670	1.046	0.924	1.185	0.479
		Place of birth: Sweden	1.030	0.993	1.068		1.085	1.048	1.124		1.095	1.056	1.135	
		Sex: Men	1.014	0.960	1.071	0.473	1.065	1.011	1.123	0.296	1.079	1.022	1.139	0.324
		Sex: Women	1.029	0.983	1.077		1.072	1.026	1.119		1.074	1.026	1.124	
	2015	Crude	1.084	1.049	1.120		1.185	1.148	1.223		1.158	1.120	1.198	
		Adjusted	1.049	1.015	1.084		1.104	1.069	1.140		1.077	1.041	1.115	
		Multivariate	1.032	0.997	1.068		1.066	1.031	1.103		1.048	1.011	1.087	
		Cohabiting: Cohabiting	1.018	0.969	1.070	0.196	1.028	0.977	1.081	0.002	1.032	0.979	1.088	0.007
		Cohabiting: Living alone	1.054	1.005	1.105		1.110	1.061	1.161		1.081	1.029	1.135	
		Place of birth: Abroad	1.024	0.919	1.141	0.651	1.102	0.988	1.229	0.900	1.014	0.904	1.136	0.252
		Place of birth: Sweden	1.052	1.015	1.089		1.107	1.069	1.145		1.086	1.047	1.126	
		Sex: Men	1.030	0.978	1.084	0.340	1.090	1.037	1.146	0.327	1.062	1.007	1.119	0.229
		Sex: Women	1.052	1.007	1.099		1.094	1.048	1.141		1.071	1.023	1.122	
	2019	Crude	1.054	1.019	1.090		1.202	1.169	1.237		1.120	1.082	1.159	
		Adjusted	1.042	1.007	1.078		1.118	1.086	1.151		1.071	1.035	1.109	
		Multivariate	1.035	0.999	1.073		1.074	1.042	1.107		1.042	1.005	1.081	
		Cohabiting: Cohabiting	1.038	0.987	1.091	0.936	1.060	1.012	1.111	0.168	1.031	0.978	1.086	0.046
		Cohabiting: Living alone	1.039	0.988	1.092		1.092	1.050	1.135		1.072	1.021	1.126	
		Place of birth: Abroad	1.040	0.940	1.151	0.951	1.054	0.960	1.156	0.141	0.996	0.899	1.103	0.136
		Place of birth: Sweden	1.042	1.004	1.081		1.128	1.094	1.164		1.080	1.041	1.121	
		Sex: Men	1.025	0.974	1.078	0.440	1.100	1.052	1.151	0.376	1.067	1.013	1.123	0.573
		Sex: Women	1.048	1.001	1.097		1.107	1.066	1.150		1.057	1.009	1.107	
